# Piezoelectric Hysteresis Modeling of Hybrid Driven Three-Dimensional Elliptical Vibration Aided Cutting System Based on an Improved Flower Pollination Algorithm

**DOI:** 10.3390/mi12121532

**Published:** 2021-12-09

**Authors:** Xifeng Fu, Hong Gong, Mingming Lu, Jiakang Zhou, Jieqiong Lin, Yongsheng Du, Ruiqi Zhou

**Affiliations:** Key Laboratory of Micro/Nano and Ultra-Precision Manufacturing of Jilin Province, School of Mechatronic Engineering, Changchun University of Technology, Changchun 130012, China; fxf201418@163.com (X.F.); zhoujiakang07@163.com (J.Z.); linjieqiong@ccut.edu.cn (J.L.); dys19961015@163.com (Y.D.); Z16688213919@163.com (R.Z.)

**Keywords:** three-dimensional elliptical vibration cutting, piezoelectric hysteresis, Bouc–Wen model, flower pollination algorithm, dynamic switching probability strategy, parameter identification

## Abstract

Three-dimensional elliptical vibration assisted cutting technology has been widely used in the past few years. The piezoelectric stack drive structure is an important part of the three-dimensional elliptical vibration aided cutting system. Its piezoelectric hysteresis characteristics affects the final output of the elliptical trajectory. Aiming at this problem, a piezoelectric hysteresis modeling method based on a generalized Bouc–Wen model is presented in this paper. An improved flower pollination algorithm (IFPASO) was used to identify Bouc–Wen model parameters. Standard test result shows that IFPASO has better algorithm performance. The model identification effect experiment proved that the Bouc–Wen model obtained by IFPASO identification, the highest modeling accuracy of the three axial subsystems, can reach 98.86%. Therefore, the model can describe the piezoelectric hysteresis characteristics of the three axial subsystems of the 3D-EVC system effectively and has higher modeling accuracy and fitting accuracy.

## 1. Introduction

With the rapid development of precision and ultra-precision machining technology, elliptical vibration cutting technology has the advantages of reducing cutting force, suppressing burrs and extending tool life. Since the end of the 1980s, this technology has received extensive attention from many experts and scholars. In order to improve the processing problems encountered by traditional cutting methods in processing certain difficult-to-machine materials, Shamoto and Moriwaki [1] proposed elliptical vibration cutting based on one-dimensional vibration cutting, namely, two-dimensional elliptical vibration cutting (EVC). The working principle is that the tool makes an elliptical movement in an orthogonal plane perpendicular on the machined surface. After adopting the elliptical vibration cutting method for difficult-to-machine materials, the mechanical machinability of such difficult-to-machine materials is improved and the cutting force and cutting heat can be reduced as much as possible during the cutting process and tool wear can be reduced. Therefore, this technology greatly improves the surface processing quality.

In the past few years, experts and scholars have made much progress and discoveries in EVC. Kim et al. [2] have done relevant research on difficult-to-machine materials and cutting shapes in elliptical vibration cutting and conducted machining experiments; Zhang C et al. [3] established a mechanical analysis model and also conducted cutting experiments on ceramic materials, which proved the effectiveness of elliptical vibration cutting. On this basis, experts and scholars proposed three-dimensional elliptical vibration cutting (3D-EVC) on the basis of two-dimensional elliptical vibration cutting and divided it into resonant type and non-resonant type. Lu et al. [4,5] studied the processing of Ti-6Al-4V alloy using a non-resonant 3D-EVC device and compared three processing methods including traditional cutting methods, thusproving the non-resonant 3D-EVC technology has better processability; Lu et al. [6] used an improved memetic algorithm to identify the nonlinear system of the three-dimensional elliptical vibration cutting system. Lin et al. [7] modeled and analyzed chip formation and transient cutting force during elliptical vibration cutting and their calculation results proved its feasibility. Compared with one-dimensional vibration cutting and EVC, 3D-EVC not only has the advantages of suppressing sharp tool wear, suppressing tool brittleness and obtaining excellent machining quality, but also obtaining higher machining efficiency. At the same time, 3D-EVC is also more beneficial to the outflow of chips and has a series of advantages of reducing friction wear between tool and workpiece.

However, there are few studies on the hysteresis and nonlinear characteristics of the piezoelectric stack structure in the non-resonant 3D-EVC system. The piezoelectric stack structure is composed of piezoelectric ceramic material and the piezoelectric ceramic material has piezoelectric hysteresis characteristics. Therefore, the inherent hysteresis and nonlinearity of the piezoelectric stack structure will directly affect the performance of the 3D-EVC device, which will also reduce the accuracy of the control system and cause instability within the device. Therefore, it is necessary to select an appropriate piezoelectric hysteresis model to describe the relationship between the axial displacement and the input voltage of 3D-EVC for accurate parameter identification.

At present, experts and scholars have proposed many mathematical models to describe the nonlinear phenomenon of piezoelectric hysteresis. For example: Arindam Bhattacharjee et al. [8] use the Preisach model, which mainly uses multiple Preisach operators and weighted superposition to describe the hysteresis characteristics; Zhou et al. [9] use the KP model to describe and model the hysteresis characteristics of piezoelectric materials; Kim et al. [10] used the Bouc–Wen model to describe the relationship between the restoring force and displacement of the hysteresis system to describe the piezoelectric hysteresis characteristics; Naser M F et al. [11] used the Duhem model, which has clear equations. The hysteresis nonlinearity can be described by adjusting the parameters of the equation; Qing et al. [12] proposes a PI mathematical model based on the improvement of the traditional Preisach model to describe the hysteresis characteristics of the piezoelectric actuator. In summary, there are many models used to describe the hysteresis characteristics, but in face of complex mathematical modeling and in order to better describe the piezoelectric hysteresis nonlinearity of the 3D-EVC system, it is necessary to choose an effective and simple hysteresis model.

Bouc–Wen model is a typical mathematical model that uses differential equations to describe hysteresis. With the continuous research of the hysteresis characteristics of piezoelectric actuators by experts and scholars, Bouc–Wen model has been gradually applied and studied with its concise and intuitive expression.

There are many parameter identification methods of Bouc–Wen model. Nowadays, experts adopt various types of intelligent algorithms to identify the parameters of this model. For example, Rakotondrabe et al. [13] proposed a method to identify the model parameters by using nonlinear filtering system; Charalampakis et al. [14] proposed an improved particle swarm optimization algorithm to identify the model parameters; Fujii et al. [15]. used the least square algorithm to identify the parameters of the improved model. It can be seen that there are many parameter identification methods for the Bouc–Wen model and they are all carried out on the basis of certain improvements to improve the identification ability.

Yang et al. [16] proposed a flower pollination algorithm in 2012. As a new type of meta-heuristic algorithm, due to its simple structure parameters and strong optimization ability, it has recently received attention from many experts and scholars. However, the flower pollination algorithm still contains the problems of early maturity and poor convergence performance of the traditional algorithm. Therefore, it is necessary to improve the flower pollination algorithm. However, there has been relatively little research on it and its application in practical problems. Nabil et al. [17] proposed a hybrid clonal selection algorithm for flower pollination algorithm to improve the performance of the algorithm; Abdel-Basset et al. [18] proposed an improved version of a cross-based flower pollination algorithm to solve the multidimensional knapsack problem; Fouad et al. [19] improved the algorithm by improving the global orientation and the best solution vector; Yang et al. [20] used a two-way learning strategy and a greedy strategy to improve the algorithm; Chen et al. [21] proposed an innovative flower pollination algorithm based on cloud mutation.

In this study, the Bouc–Wen model will be used to describe the hysteresis nonlinearity exhibited by the 3D-EVC system and an improved flower pollination algorithm will be used to identify the parameters of the model. The improved flower pollination algorithm introduces the early particle swarm optimization and dynamic switching probability strategy to improve the accuracy of model parameter identification and uses the standard test function to test the algorithm performance. Finally, the identification of the model parameters and the verification of the identification effect are carried out.

## 2. Establishment of Piezoelectric Hysteresis Model for Three-Dimensional Elliptical Vibration Cutting (3D-EVC)

For the non-resonant 3D-EVC device, the realization of its final elliptical trajectory output is achieved through the piezoelectric driver output displacement and then through the structural transmission and synthesis. The target system studied in this paper is a stack type piezoelectric actuator. Similarly, as a piezoelectric material, the hysteresis characteristic is part of its inherent nature. Its main manifestation is that the rising voltage-driven output displacement curve applied to the piezoelectric material does not completely coincide with the drop voltage-driven output displacement curve, thus forming a hysteresis loop. Since the piezoelectric stack is a vital component in the entire non-resonant 3D-EVC system, the piezoelectric hysteresis characteristics of the piezoelectric material will seriously affect the output of the final elliptical trajectory and the control of the entire processing system accuracy. In summary, in this section, we will consider the use of a piezoelectric hysteresis model to describe the axial motion of the non-resonant 3D-EVC system.

### 2.1. The Structure of the 3D-EVC System

The research in this paper is based on a self-designed non-resonant three-dimensional elliptical vibration aided cutting system, which is driven by three piezoelectric stacks with a “two parallel and one vertical” positional relationship, which is mainly composed of two flexible systems perpendicular to each other composition. The system can adjust various processing parameters in three-dimensional elliptical vibration cutting, thereby obtaining higher and good processing performance.

**Figure 1:**
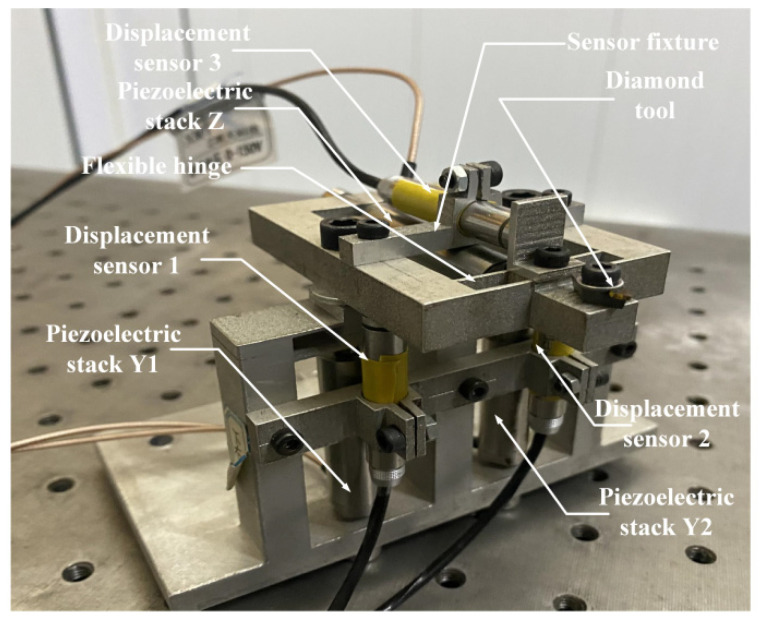
The structure of the 3D-EVC system.

The specific structure of the system is shown in Figure 1. Three piezoelectric stacks are respectively distributed on the upper flexible hinge and the lower flexible hinge and each piezoelectric stack is placed in parallel with a displacement sensor. Each piezoelectric stack in a single direction will drive the corresponding flexible hinge to produce slight deformation after receiving a certain signal drive, thereby driving the entire 3D-EVC system to produce various axial displacements. There is a certain phase difference between the drive signals in each direction, so the axial displacement generated by these drives will work together to promote the tool tip to form a three-dimensional elliptical motion track, so as to achieve the purpose of three-dimensional elliptical motion assisted cutting.

It can be known from the working principle of the 3D-EVC system that the unidirectional sub-motion of the 3D-EVC system is the relationship between the input voltage and the output displacement of the piezoelectric stack actuator. This nonlinear relationship between voltage and displacement can be used as a piezoelectric hysteresis model to describe. In this paper, we will choose the Bouc–Wen model to describe.

### 2.2. Bouc–Wen Model

The Bouc–Wen model is a phenomenological mathematical model that uses differential equations to describe hysteresis. Compared with other operator-based models, the Bouc–Wen model has fewer parameters and a more expressive form, which exists as a form of nonlinear differential equations. For the intuitive advantage, it can not only characterize the mathematical characteristics of hysteresis, but also describe the dynamic characteristics of the piezoelectric actuator. It is precisely because the Bouc–Wen model has the ability to simulate various hysteresis behaviors, it is widely used in structural materials and systems with hysteresis franchise.

The early Bouc–Wen model is a basic and simple form of first-order differential equation [22]. In recent years, with the research on smart material drive mechanisms, the Bouc–Wen model has gradually been used to describe the hysteresis and nonlinear characteristics of smart material actuators such as piezoelectric ceramic actuators and giant magnetostrictive actuators [13]. The mathematical expression of the Bouc–Wen model commonly used at present is represented by the following equation:

(1) $$\left\{ \begin{array}{l} Mx^{\prime \prime }(t)+Bx^{\prime }(t)+kx(t)=C[Du(t)-h(t)]\\ h^{\prime }(t)=Au^{\prime }(t)-\beta \left|u^{\prime }(t)\right|h(t){\left|h(t)\right|}^{n-1},\;\;h(0)=0 \end{array}\right.$$

Considering that in this paper, the Bouc–Wen model describes the hysteresis characteristics based on the non-resonant 3D-EVC. Therefore, both from the experimental point of view and the identification in the next chapter, they are all performed at low frequencies. Therefore, $Mx^{\prime \prime }(t)$ in Equation (1) can be ignored. At the same time, the initial displacement x(t) can be processed by recalibrating the displacement sensor. Based on the above factors and at the same time to facilitate subsequent identification, we simplified and organized the structure of the Bouc–Wen model to get the following equation:

(2) $$\left\{ \begin{array}{l} x^{\prime }(t)=c_{0}+c_{1}u(t)+c_{2}h(t)\\ h^{\prime }(t)=Au^{\prime }(t)-\beta \left|u^{\prime }(t)\right|h(t){\left|h(t)\right|}^{n-1}-\gamma u^{\prime }(t){\left|h(t)\right|}^{n}\\ y(t)=x(t) \end{array}\right.$$

In summary, in order to use the simplified Bouc–Wen model to describe the piezoelectric hysteresis behavior, we need to use a suitable and excellent algorithm to identify some unknown parameters in the model. From Equation (2), we can see that the identification of parameter is $c_{0},c_{1},c_{2},A,\beta ,\gamma ,n$. The specific identification methods and identification results will be introduced in the subsequent chapters.

## 3. Improved Flower Pollination Algorithm (IFPASO)

As we all know, even the most advanced and complete algorithms cannot produce the most satisfactory results for all optimization problems. Flower pollination algorithm is a meta-heuristic algorithm proposed in recent years. It has the advantages of easy implementation, fewer parameters and strong optimization ability.

However, the flower pollination algorithm also has some shortcomings of the traditional meta-heuristic algorithm, such as the low accuracy of the later optimization and the shortcomings of being easy to fall into local extreme values. For the above defects existing in FPA, this paper will improve the traditional FPA.

### 3.1. Flower Pollination Algorithm (FPA)

Inspired by the flower pollination process of flowering plants, Yang proposed a new swarm intelligence optimization algorithm in 2012 to solve related practical problems and named the intelligent optimization algorithm as flower pollination algorithm [16].

Flower pollination algorithms need to be based on the following types of premises:

(1) Biological cross-pollination is considered to be a global pollination process and pollinators use Lévy flight to pollinate;
(2) Non-biological self-pollination is considered local pollination;
(3) Flower constancy is considered to be the probability of reproduction, which is proportional to the similarity of the two flowers participating in pollination;
(4) The conversion between local pollination and global pollination is controlled by the transition probability p∈[0,1]. Due to the influence of physical conditions and other factors, local pollination should have a significant bias p in the overall pollination process.

In order to better express the above rules, we will express the above rules in the form of mathematical formulas. For example, in the global pollination stage, pollen is carried and spread by pollinators such as insects, because pollinators can carry pollen in a larger search range, so pollen can be spread over a longer distance. Equation (3) is used to express flowers’ global pollination and flower constancy:

(3) $$x_{i}^{t+1}=x_{i}^{t}+\gamma L(\lambda )(x_{i}^{t}-g_{best}^{*})$$

L(λ) is the parameter corresponding to the pollination intensity, that is, the flight step length. Since pollinators may move long distances in different steps, Lévy flight can be used to effectively express this feature, L(λ) is expressed by Equation (4) and L(λ)>0:

(4) $$L(\lambda )∼\frac{\lambda \Gamma (\lambda )\sin(\pi \lambda /2)}{\pi }\frac{1}{S^{1+\lambda }},(S>S_{0}>0)$$

According to the Mantegna algorithm, S can be described by two Gaussian distributions of U,V:

(5) $$S=\frac{U}{{\left|V\right|}^{1/\lambda }},\;U\sim N(0,\sigma^{2}),\;V\sim N(0,\sigma^{2})$$

(6) $$\sigma^{2}={\left\{\frac{\Gamma (1+\lambda )}{\lambda \Gamma [(1+\lambda )/2}\cdot \frac{\sin(\pi \lambda /2)}{2^{(\lambda -1)/2}}\right\}}^{1/\lambda }$$

Under the assumptions of rules (3) and (4), local pollination can be expressed by Equation (7):

(7) $$x_{i}^{t+1}=x_{i}^{t}+\varepsilon (x_{j}^{t}-x_{k}^{t})$$

The pseudo-code of the standard flower pollination algorithm is shown as following Algorithm 1:
**Algorithm 1.** Standard flower pollination algorithm.1: Define the objective function $f(x),x=(x_{1},x_{2},\ldots ,x_{d})$ 2: Initialize a population of n flowers/pollen gametes with random solutions 3: Evaluate each flower or solution in the population 4: Extract the best solution in the population 5: Find the best solution $g_{best}^{*}$ in the initial population 6: Define a switch probability p∈[0,1] 7: Define fixed number of iterations Max_generation 8: **While** t<Max_generation 9: **for** i = 1:n (each flower in the population) 10: **if** (rand < p) 11: Draw a (d-dimensional) step vector L which obeys a Lévy distribution 12: Global pollination via $x_{i}^{t+1}=x_{i}^{t}+\gamma L(\lambda )(x_{i}^{t}-g_{best}^{*})$ 13: **else** 14: Draw ε from a uniform distribution in [0, 1] 15: Do local pollination via $x_{i}^{t+1}=x_{i}^{t}+\varepsilon (x_{j}^{t}-x_{k}^{t})$ 16: **end if** 17: Evaluate each new solution $x_{i}^{t}$ 18: **If** new solution is better, update it in the population 19: **end for** 20: Find the current best solution $g_{best}^{*}$ 21: **end While** 22: Output the best solution found

### 3.2. Dynamic Switching Probability Strategy

In FPA, the local search and global search are adjusted by the conversion probability, which is a fixed value in the standard flower pollination algorithm. However, during the whole process of the algorithm operation, we prefer to perform more global searches at the beginning of the search to expand the search space and to enhance the execution of local searches in the later stage to speed up the speed of finding the best solution. By introducing a dynamic switching probability strategy in the later stage, the algorithm can adaptively adjust the ratio of local search and global search, so that the algorithm is no longer easy to fall into the range of partial optimal values when searching for optimization. Therefore, we adopt a dynamic conversion probability strategy to adjust the proportion of global search and local search in the entire search process. The switching probability is expressed by Equation (8):

(8) $$0.8-0.1*\frac{Max\_T-t}{Max\_T}$$

### 3.3. Early-Stage Particle Swarm Optimization

The initial solution of the algorithm plays a vital role in the quality of the optimization results and the initial solution of the FPA algorithm is generated randomly in the feasible region. When the value of one of the solutions deviates too much from the theoretical optimal value, it not only increases the search difficulty of the algorithm, but also greatly affects the convergence speed of the algorithm. Particle swarm optimization (PSO) is a search algorithm used to solve optimization in computational mathematics and it is also one of the most classic intelligent algorithms [23]. The goal of particle swarm optimization is to make all particles find the optimal solution in a multi-dimensional hyper-volume [24].

Suppose that in a D-dimensional target search space, there is a particle population with a population size of N, that is, there are a total of particles in the population. The position of the *i*-th particle is expressed as an N-dimensional vector $X_{i}=(x_{i1},x_{i2},\ldots x_{iD})(i=1,2\ldots N)$ and its flight speed can be expressed as $Vi=(v_{i1},v_{i2},\ldots v_{iD})(i=1,2,\ldots N)$. The position of each particle represents a feasible solution to a problem in the target search space. At the beginning of the algorithm, the population is initialized as a set of random solutions, that is, randomly distributed in the entire search space. When the algorithm is executed, the state of the particles is updated mainly through Equations (9) and (10):

(9) $$v_{t+1}^{i}=w_{t}v_{t}^{i}+c_{1}r_{1}(p_{t}^{i}-x_{t}^{i})+c_{2}r_{2}(p_{t}^{g}-x_{t}^{i})$$

(10) $$x_{t+1}^{i}=x_{t}^{i}+v_{t+1}^{i}$$

where $w_{t}$ is the inertia weight of the particle, the larger the value, the stronger the particle’s exploration ability;

(11) $$w_{t}=(w_{\max}-w_{\min})*\frac{\left(t_{\max}-t\right)}{t_{\max}}+w_{\min}$$

The pseudo code of the particle swarm algorithm is given as following Algorithm 2:
**Algorithm 2.** Particle swarm algorithm.1: **Start** 2: Randomly initialize particle swarm 3: **While** (number of iterations or the stopping iteration on is not met) 4: Evaluate fitness of the particle swarm 5: **for** n = 1 to number of particles 6: Find individual optimal solution $p_{t}^{i}$ 7: Find group optimal solution $p_{t}^{g}$ 8: **for** d = 1 to number of dimensions of particle 9: update the velocity of particles via $v_{t+1}^{i}=w_{t}v_{t}^{i}+c_{1}r_{1}(p_{t}^{i}-x_{t}^{i})+c_{2}r_{2}(p_{t}^{g}-x_{t}^{i})$ 10: update the position of particles via $x_{t+1}^{i}=x_{t}^{i}+v_{t+1}^{i}$ 11: **end for** 12: **end for** 13: update the inertia weight via $w_{t}=(w_{\max}-w_{\min})*\frac{\left(t_{\max}-t\right)}{t_{\max}}+w_{\min}$ 14: **end While** 15: Output the best solution found

Therefore, we introduce PSO in the early stage of FPA execution to compensate for the randomness generated by the initial solution in FPA. In the IFPASO execution process, it is divided into two stages, the first stage executes PSO and the second stage executes FPA. Through this method, we can make the search range closer to the area where the optimal solution is located and avoid the possibility of invalid value divergence, thereby increasing the algorithm’s optimization ability and its convergence speed.

In summary, based on the dynamic conversion probability strategy and the introduction of PSO in the early stage of FPA execution, the algorithm’s ability to solve practical problems can be greatly improved. The pseudo code of the IFPASO algorithm is shown as follows Algorithm 3:
**Algorithm 3.** IFPASO algorithm.1: **Start** 2: Randomly initialize particle swarm 3: **While** (number of iterations or the stopping iteration on is not met) 4: Evaluate fitness of the particle swarm 5: **for** n = 1 to number of particles 6: Find individual optimal solution $p_{t}^{i}$ 7: Find group optimal solution $p_{t}^{g}$ 8: **for** d = 1 to number of dimensions of particle 9: update the velocity of particles via $v_{t+1}^{i}=w_{t}v_{t}^{i}+c_{1}r_{1}(p_{t}^{i}-x_{t}^{i})+c_{2}r_{2}(p_{t}^{g}-x_{t}^{i})$ 10: update the position of particles via $x_{t+1}^{i}=x_{t}^{i}+v_{t+1}^{i}$ 11: **end for** 12: **end for** 13: update the inertia weight via $w_{t}=(w_{\max}-w_{\min})*\frac{\left(t_{\max}-t\right)}{t_{\max}}+w_{\min}$ 14: **end While** 15: Output the best solution found 16: The best solution found by PSO is regarded as initial points for FPA algorithm $g_{best}^{*}$ 17: **While** t<Max_generation 18: **for** i=1:n (each flower in the population) 19: get dynamic switch probability p via $0.8-0.1*\frac{Max\_T-t}{Max\_T}$ 20: **if** (rand < p) 21: Draw a (d-dimensional) step vector L which obeys a Lévy distribution 22: Global pollination via $x_{i}^{t+1}=x_{i}^{t}+\gamma L(\lambda )(x_{i}^{t}-g_{best}^{*})$ 23: **else** 24: Draw ε from a uniform distribution in [0, 1] 25: Do local pollination via $x_{i}^{t+1}=x_{i}^{t}+\varepsilon (x_{j}^{t}-x_{k}^{t})$ 26: **end if** 27: Evaluate each new solution $x_{i}^{t}$ 28: **If** new solution is better, update it in the population 29: **end for** 30: Find the current best solution $g_{best}^{*}$ 31: **end While** 32: Output the final best solution found

### 3.4. IFPASO Performance Test

In order to verify the effectiveness of the proposed algorithm, we compare IFPASO with traditional FPA and PSO algorithms for verification. We have selected two well-known benchmark function functions for verification here. For the two algorithms, for comparison and verification, we have adopted the most common parameter settings in the literature. For fairness, we use the same fixed individual scale for the above algorithms and all the algorithms are independently run the same number of times, the following is the introduction of the two selected benchmark functions:

Test function 1: Ackle() function:

(12) $$f(x)=-20\exp\left(-0.2\sqrt{\frac{1}{n}\sum_{j=1}^{n}x_{j}^{2}}\right)-\exp\left(\frac{1}{n}\sum_{j=1}^{n}\cos(2\pi x_{j})\right)+20+e-8\leq x\leq 8$$

Test function 2: Schaffer() function:

(13) $$\min f(x_{1},x_{2})=0.5+\frac{{\left(\sin\sqrt{x_{1}^{2}+x_{2}^{2}}\right)}^{2}-0.5}{{\left(1+0.001(x_{1}^{2}+x_{2}^{2})\right)}^{2}}-10.0\leq x_{1},x_{2}\leq 10.0$$

**Figure 2:**
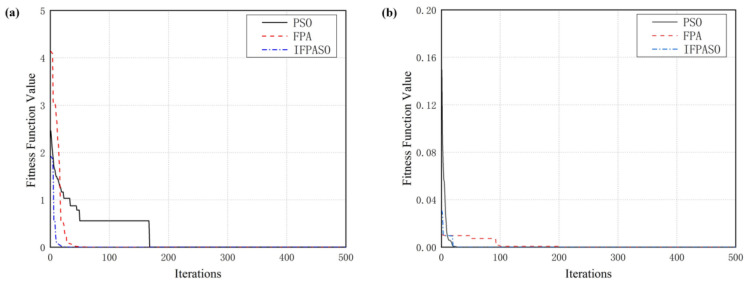
Convergence result (**a**) Ackle() function (**b**) Schaffer() function.

Figure 2 is the test result of the Ackle() function and the Schaffer() function using PSO, FPA and IFPASO respectively.

Table 1 shows the best values of PSO, FPA and IFPASO for the final results of the Ackle() function and Schaffer() function.

It is seen from the iterative curve and convergence optimum results that although the three algorithms converge to the global optimal solution, IFPASO shows faster convergence speed and higher accuracy for both test functions. As a result, it can be concluded that the IFPASO proposed in this paper has excellent optimization ability and overcomes some drawbacks of traditional algorithms to some extent. Therefore, we can use IFPASO for the parameter identification of Bouc–Wen model.

## 4. Simulation Experiment and Result Analysis

In this section, the IFPASO algorithm with good optimization performance verified above is applied to the parameter identification of the Bouc–Wen model and, finally, the accuracy of the identification results is verified through the modeling comparison in 3D-EVC.

### 4.1. Experimental Setup

The experimental setup part is mainly divided into hardware part and software part. Hardware part: 3D-EVC system, PC, signal generator, power amplifier, displacement sensor, Power PMAC; Software part: Matlab2012a.

**Figure 3:**
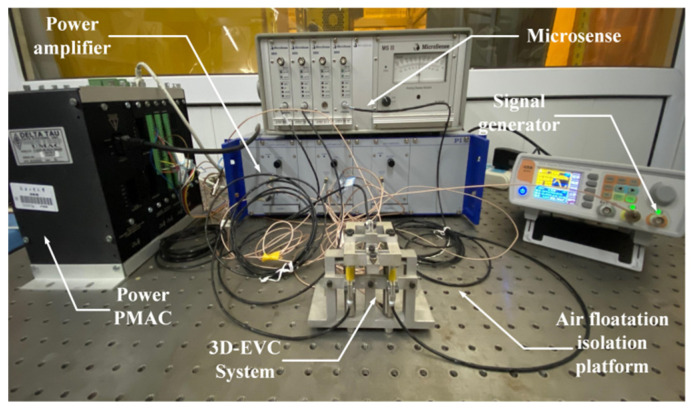
Experimental setup.

The Bouc–Wen model parameter identification experiment setting of 3D-EVC system is shown in Figure 3.

### 4.2. Parameter Identification of Bouc–Wen Model

According to the Bouc–Wen dynamic nonlinear model we established in the previous content, we can see that the parameters we need to identify are $c_{0},c_{1},c_{2},A,\beta ,\gamma ,n$. Based on the piezoelectric hysteresis characteristics of the 3D-EVC system and the piezoelectric stack, in order to identify the parameters in the Bouc–Wen model, a sinusoidal excitation signal is given to the 3D-EVC system for parameter identification of the piezoelectric hysteresis model.

**Figure 4:**
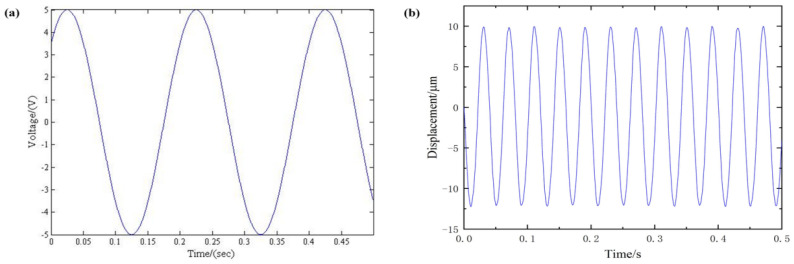
Excitation condition (**a**) sinusoidal excitation signal (**b**) corresponding displacement excitation curve.

In order to identify the parameters in the Bouc–Wen model used to characterize the Y1 axial subsystem, a sinusoidal excitation signal is given to the 3D-EVC system, as shown in Figure 4 and the corresponding displacement excitation curve collected by the displacement sensor.

The results of the Bouc–Wen model parameter identification of each axial subsystem of the 3D-EVC system are shown in the Table 2:

### 4.3. Test of Model Identification Effect

In order to verify whether the Bouc–Wen model obtained through parameter identification can accurately describe the hysteresis nonlinearity exhibited by the 3D-EVC system, the hysteresis curve output under the Bouc–Wen model was fitted with the actual hysteresis curve under 50 Hz excitation and the fitting error was calculated. In this paper, the mean square error (MSE) is selected as the target fitness function for model accuracy verification and its expression is shown in Equation (14):

(14) $$OF(r)=\frac{1}{N}\sum_{i=1}^{N}(x_{\exp}(i)-x_{mdl}(i){)}^{2}$$

**Figure 5:**
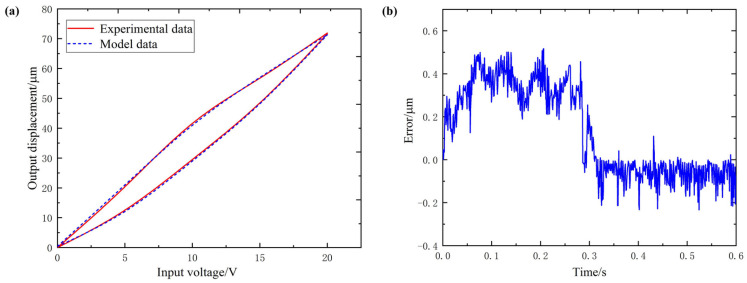
(**a**) hysteresis curve fitting of Y1 (**b**) fitting error of Y1.

**Figure 6:**
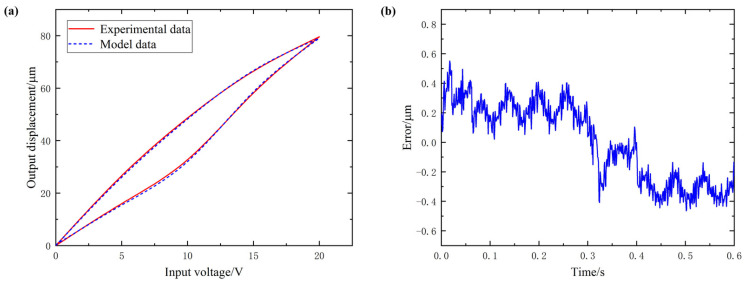
(**a**) hysteresis curve fitting of Y2 (**b**) fitting error of Y2.

**Figure 7:**
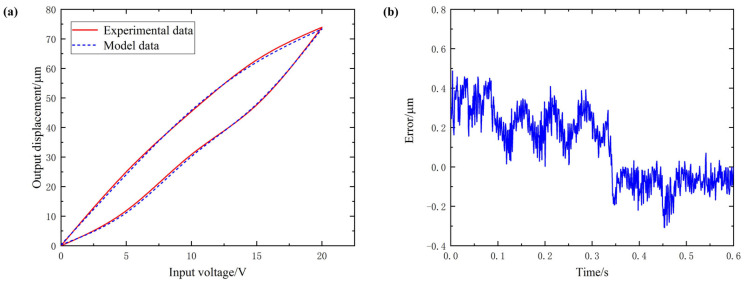
(**a**) hysteresis curve fitting of Z (**b**) fitting error of Z.

Figure 5, Figure 6 and Figure 7 are, respectively, the hysteresis curve fitting diagram and fitting error diagram of the Y1, Y2 and Z-direction subsystems of the 3D-EVC system. The maximum modeling error and modeling accuracy results of each axial subsystem are shown in Table 3.

From the hysteresis curve fitting and fitting error of the three axial subsystems, it can be seen that the Bouc–Wen model obtained by using the IFPASO algorithm proposed in this paper for parameter identification can effectively and accurately describe the hysteresis of the 3D-EVC system. The maximum modeling error is only 0.5332 μm and the maximum modeling accuracy can reach 98.86%, which can satisfy the accuracy requirements of subsequent related work for hysteresis modeling and it has high modeling accuracy.

## 5. Conclusions

This paper focuses on the 3D-EVC system due to the piezoelectric hysteresis characteristics that affect the precision machining accuracy and the output of the elliptical trajectory. A Bouc–Wen hysteresis model is used to characterize the relationship between the input voltage and the output displacement of the 3D-EVC system.

1. In order to improve the accuracy of model parameter identification, this paper proposes an improved flower pollination algorithm (IFPASO) based on the original flower pollination algorithm (FPA) algorithm structure, introducing particle swarm optimization (PSO) and dynamic conversion probability strategy. Performance test results show that the new flower pollination algorithm (IFPASO) has better optimization effect and faster convergence speed.
2. The results of the model identification effect test show that the Bouc–Wen model obtained by using the new flower pollination algorithm (IFPASO) identification parameters can completely describe the piezoelectric hysteresis characteristics of the three axial subsystems of the 3D-EVC system and has high Modeling accuracy.
3. The maximum modeling error and modeling accuracy of the three axial subsystems Y1, Y2 and Z are 0.5074 μm, 98.24%; 0.5332 μm, 98.05%; 0.4878 μm, 98.86%, respectively. It can be seen that the Bouc–Wen model obtained by using the improved flower pollination algorithm (IFPASO) for parameter identification can effectively characterize the piezoelectric hysteresis characteristics of the 3D-EVC system and the fitting accuracy is higher. It provides a theoretical model reference and basis for the control system design of the high-performance 3D-EVC system.

## Figures and Tables

**Table 1:** Comparison results of the three algorithms.

	PSO	FPA	IFPASO
Ackle () function	0.000780527	5.35228 × 10^−5^	1.00 × 10^−8^
Schaffer () function	5.39183 × 10^−5^	4.13 × 10^−9^	1.00 × 10^−15^

**Table 2:** Bouc–Wen model parameters of each axis of 3D-EVC system.

	$C_{0}$	$C_{1}$	$C_{2}$	A	B	γ	n
Y1	−1349.77	1863.74	−1543.57	0.3961	0.5765	0.21	1.2
Y2	−1479.62	1938.74	−1637.54	0.3879	0.6524	0.2483	1
Z	−1551.36	1926.21	−1703.45	0.4215	0.6952	0.2431	1

**Table 3:** Modeling error and accuracy of each axis of 3D-EVC system.

	Maximum Modeling Error	Modeling Accuracy
Y1	0.5074 μm	98.24%
Y2	0.5332 μm	98.05%
Z	0.4878 μm	98.86%

## Data Availability

Not applicable.

## Nomenclature

M	the system’s mass	Γ(λ)	standard gamma function
B	damping coefficient	$x_{j}^{t},x_{k}^{t}$	random pollen
K	stiffness	ε	random number in [0, 1]
C	scale factor	p	switching probability
x(t)	state variable	$v_{t}^{i}$	particle i speed at iteration t
h(t)	output of the hysteresis part	$p_{t}^{i}$	particle i optimal solution
D	ratio coefficient	$p_{t}^{g}$	population optimal solution
A,B	hysteresis parameter	$c_{1},c_{2}$	learning factor
γ,n	hysteresis parameter	$r_{1},r_{2}$	random number in [0, 1]
u(t)	input signal	$w_{t}$	inertia weight
$u^{\prime }(t)$	first derivative of u(t)	$w_{\min}$	minimum value of $w_{t}$
$c_{0},c_{1},c_{2}$	coefficient to be identified	$w_{\max}$	maximum value of $w_{t}$
y(t)	output displacement of the system	$t_{\max}$	maximum number of iterations
$x_{i}^{t}$	solution i at iteration t	Max_T	maximum number of iterations
$x_{i}^{t+1}$	solution i at iteration t+1	t	current iteration number
$g_{best}^{*}$	global optimal solution	r	$r=(c_{0},c_{1},c_{2},A,\beta ,\gamma ,n)$
γ	scale factor of the control step	N	total number of data
L(λ)	Lévy flight	$x_{\exp}$	experimental data
S	step length	$x_{mdl}$	model data
